# Description of three new species of *Arescon* Walker (Hymenoptera, Mymaridae) from China

**DOI:** 10.3897/zookeys.584.8129

**Published:** 2016-04-25

**Authors:** Xiang-Xiang Jin, Cheng-De Li, Jian-Chun Yang

**Affiliations:** 1 Guangdong Entomological Institute, Guangzhou, 510260, China; 2 Guangdong Public Laboratory of Wild Animal Conservation and Utilization, Guangzhou, 510260, China; 3 Guangdong Key Laboratory of Integrated Pest Management in Agriculture, Guangzhou, 510260, China; 4 School of Forestry, Northeast Forestry University, Harbin, 150040, China

**Keywords:** Chalcidoidea, Mymaridae, Arescon, taxonomy, new species, China

## Abstract

Three new species of *Arescon* Walker, 1846, *Arescon gaoligongensis* Jin & Li, **sp. n.**, *Arescon sparsiciliatus* Jin & Li, **sp. n.** and *Arescon stenopterus* Jin & Li, **sp. n.** are described. A key to the Chinese species is given and photomicrographs are provided to illustrate morphological characters. All the specimens are deposited in the insect collections of Northeast Forestry University, China.

## Introduction

*Arescon* currently contains 22 species according to Noyes (2015). Among them, *Arescon armata* (Meunier, 1906) and *Arescon baltica* (Meunier, 1901) are fossils; *Arescon aspidioticola* (Ashmead, 1879) and *Arescon peregrina* (Perkins, 1910) are *nomina dubia* according to Schauff (1984) and Beardsley and Huber (2000), respectively. The type material of both species is lost; the Ashmead species likely belongs to Aphelinidae (Schauff 1984) and the Perkins species probably does not belong to *Arescon* but its generic placement within Mymaridae is uncertain. Since 1990, only one species, *Arescon zenit* Triapitsyn & Berezovskiy, 2003, has been described as new.

In China, Lin and Xu (2000) keyed *Arescon* in their key to 19 Chinese genera of Mymaridae and briefly summarized its distribution and hosts. Tian (2009) reported *Arescon iridescens* (Enock, 1914) from Hainan Province. In this study, we describe 3 new species and provide a key to the *Arescon* species found in China.

## Material and methods

We collected 15 specimens (12 females and 3 males) of *Arescon* in Yunnan Province and Xizang Autonomous Region (= Tibet) by sweeping, Malaise traps (MT) and yellow pan traps (YPT). Specimens were dissected and mounted in Canada balsam on slides following the method described by Noyes (1982) and modified for Mymaridae by Huber (2015). Photographs were taken with a digital CCD camera attached to an Olympus BX51 compound microscope. Most measurements were made from slide-mounted specimens using an eye-piece reticle. Total body length excluding ovipositor was measured with an eye-piece reticle from ethanol-preserved specimens before being dissected. All measurements are given in micrometers (μm). All the specimens listed below are deposited in Northeast Forestry University, Harbin, China (NEFU).

Morphological terminology and abbreviations are adopted from Gibson (1997) and Huber (2012), as follows (with some additions):

Fln
 Flagellar segment 


Mps
 Multiporous plate sensilla 


### Key to *Arescon* species in China

**Table:** 

1	♀: flagellum clavate, funicle 5-segmented and clava 1-segmented	2
−	♂: flagellum filiform, 11-segmented	5
2	Fore wing with venation extending just about half length of wing (Fig. 9); metanotum with dorsellum distinctly triangular (Fig. 8)	***Arescon stenopterus* sp. n.**
−	Fore wing with venation extending about 0.6–0.8× length of wing (Figs 4, 19); metanotum with dorsellum rhomboidal (Figs 3, 18)	**3**
3	Fl_2_ longer than fl_3_; fore wing with disc densely setose, with at least 6 irregular rows of setae at broadest part of the wing (Fig. 4)	**4**
−	Fl_2_ about as long as or shorter than fl_3_ (Fig. 17); fore wing with disc sparsely setose, with at most 3 irregular rows of setae at broadest part of the wing (Fig. 19)	***Arescon sparsiciliatus* sp. n.**
4	Fore wing relatively narrow, length/width 3.9 (Fig. 4); propodeum relatively short, not more than 0.7× length of scutellum (Fig. 3)	***Arescon gaoligongensis* sp. n.**
−	Fore wing relatively broad, length/width 3.2–3.4; propodeum relatively long, about as long as scutellum	***Arescon iridescens* (Enock)**
5	Fore wing with venation extending just about half length of wing (Fig. 14); metanotum with dorsellum distinctly triangular	***Arescon stenopterus* sp. n.**
−	Fore wing with venation extending just about 0.6–0.8× length of wing (Fig. 25); metanotum with dorsellum rhomboidal (Fig. 24)	**6**
6	Fore wing disc densely setose, with at least 6 irregular rows of setae at broadest part of the wing	***Arescon iridescens* (Enock)**
−	Fore wing disc sparsely setose, with at most 3 irregular rows of setae at broadest part of the wing (Fig. 25)	***Arescon sparsiciliatus* sp. n.**

## Taxonomy

### Arescon gaoligongensis

Taxon classificationAnimaliaHymenopteraMymaridae

Jin & Li sp. n.

http://zoobank.org/06864F6D-CB04-4258-932B-F0AAE9F17A38

Figs 1–6

#### Holotype.

♀ (NEFU) Yunnan Province, Baoshan City, Mt. Gaoligong, Baihualing, 31. VII.2014–2.VIII. 2014, Hui-Lin Han, YPT.

#### Diagnosis.

Clava (Fig. 2) 2.93× as long as wide, longer than scape; metanotum (Fig. 3) with dorsellum rhomboidal; propodeum distinctly shorter than scutellum; phragma broad with posterior margin nearly straight; fore wing (Fig. 4) 3.93× as long as wide, with venation extending 0.7× length of wing; discal setation rather sparse, with about 7 or 8 rows of setae at the broadest part of wing; base of the wing behind submarginal vein asetose; ovipositor (Fig. 6) distinctly exserted, 2.12× as long as metatibia.

**Figures 1–6.**
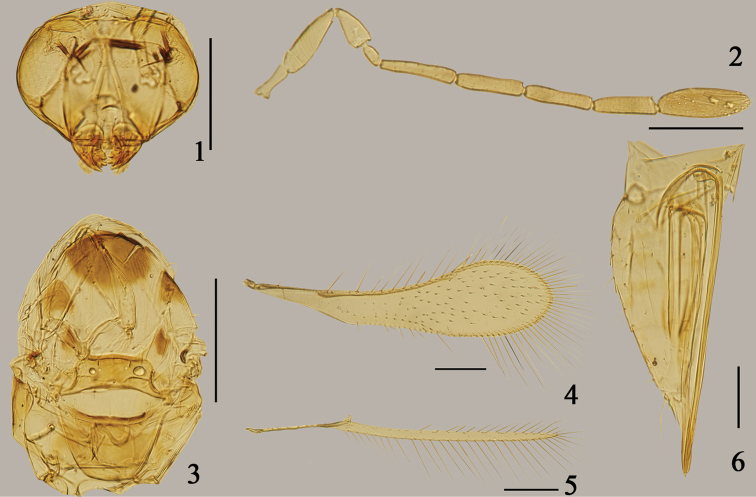
*Arescon gaoligongensis* sp. n., holotype female: **1** head, dorsal **2** antenna **3** mesosoma, dorsal **4** fore wing **5** hind wing **6** gaster, lateral. Scale bars: 100 μm.

#### Description.

Female (Holotype). Body length 756. Head yellowish brown with eye, ocelli, middle part of transverse trabecula, supraorbital trabecula and mandible dark brown. Antenna yellowish brown with radicle, scape, pedicel and fl_1_ paler. Mesosoma largely yellowish brown except a large round spot on about anterior two fifths and two relatively small spots on lateral margins of mesoscutum, a small spot on each axilla anteriorly, dark brown. Wings slightly infuscated, with venation brown. Legs brown with basal parts of coxae, apical parts of femora and last tarsal segments paler. Metasoma pale brown with exerted part of ovipositor darker.

Head. Head (Fig. 1) width 168. Vertex and face with faint reticulate sculpture.

Antenna. Antenna (Fig. 2) sparsely setose. Radicle 0.46× as long as scape; scape about 3.5× as long as wide, with distinct striations which are more or less transverse on base and gradually become oblique distad; pedicel with faint longitudinal striations, about 2× as long as wide, and 2× as long as fl_1_; all funicular segments much longer than wide, fl_1_ distinctly shortest, without mps; fl_2_ slightly longer than fl_3_, with 1 mps; fl_3_ about as long as fl_4_ each with 2 mps; fl_5_ slightly shorter than fl_4_, with 2 mps; clava 2.93× as long as wide, longer than scape, shorter than fl_4_ and fl_5_ combined, divided into 3 segments ventrally by 2 incomplete oblique septa, with 6 mps. Measurements (length/width): radicle 38, scape 84/24, pedicel 48/24, fl_1_ 24/13, fl_2_ 82/17, fl_3_ 72/17, fl_4_ 72/17, fl_5_ 67/19, clava 98/34.

Mesosoma. Mesosoma (Fig. 3) length 277. Pronotum entire, with faint longitudinal striations. Mesoscutum with longitudinal reticulate sculpture on mid lobe and isodiametric reticulate sculpture on lateral lobes. Scutellum transverse, distinctly shorter than mesoscutum (30: 51); anterior scutellum (14: 33) subrectangular, with campaniform sensilla a little nearer to lateral margin than to each other. Metanotum with dorsellum rhomboidal. Propodeum smooth, distinctly shorter than scutellum. Phragma broad with posterior margin nearly straight.

Wings. Fore wing (Fig. 4) length 584, width 149, length/width 3.93, with venation extending 0.7× length of wing; longest marginal setae 152, 1.02× as long as greatest wing width. Fore wing base behind submarginal vein without setae, disc behind basal half of marginal vein with 2 or 3 irregular rows of setae, remaining disc distal to middle of marginal vein with 7 or 8 irregular rows of setae and a bare strip present along about distal one third of posterior margin. Hind wing (Fig. 5) length 545, width 17, length/width 32.4, longest marginal setae 101, about 6× as long as greatest wing width.

Metasoma. Metasoma (Fig. 6) distinctly longer than mesosoma. Petiole short. Gaster (376) with ovipositor length 495, distinctly exserted, 2.12× as long as metatibia (233).

#### Host.

Unknown.

#### Etymology.

The specific name is derived from the name of the collection locality of the type species.

#### Comments.

*Arescon gaoligongensis* sp. n. is similar to *Arescon iridescens*, but can be distinguished from it by the key given above. The new species is also similar to *Arescon enocki* (Subba Rao & Kaur) in relatively longer fore wing venation and fore wing disc setation, but can be distinguished from it by the relatively shorter clava, 2.9× as long as wide, shorter than fl_4_ and fl_5_ combined (clava relatively longer, 4.0× as long as wide, much longer than fl_4_ and fl_5_ combined in *Arescon enocki*); broader fore wing, 3.9× as long as wide (much narrower, 4.5× as long as wide in *Arescon enocki*); and the ovipositor characters, ovipositor originated from base of gaster, distinctly exserted (ovipositor originated from distal part of gaster, and slightly exserted in *Arescon enocki*).

### Arescon stenopterus

Taxon classificationAnimaliaHymenopteraMymaridae

Jin & Li sp. n.

http://zoobank.org/15741396-703A-4F7D-9DF8-DB83FED7B4A6

Figs 7–11 , 12–16

#### Holotype.

♀ (NEFU) Xizang Autonomous Region (= Tibet), Mt. Sejila, 30.VII. 2013–01.VIII. 2013, Hui-Lin Han, Zhi-Guang Wu, YPT.

#### Paratypes.

**6 females, 1 male.** Xizang Autonomous Region (= Tibet): same data as holotype (1♀, NEFU); Linzhi City, 28.VII. 2012–04.VIII. 2012, Zhao-Hui Pan, MT (2 ♀♀, NEFU); Mt. Sejila, 27. VII. 2013, Hui-Lin Han, Zhi-Guang Wu, YPT (1♀, NEFU); Mt. Sejila, 4100 m, 22. VIII. 2014–23. VIII. 2014, Hui-Lin Han, YPT (2 ♀♀ 1♂, NEFU).

#### Diagnosis.

**Figures 7–11.**
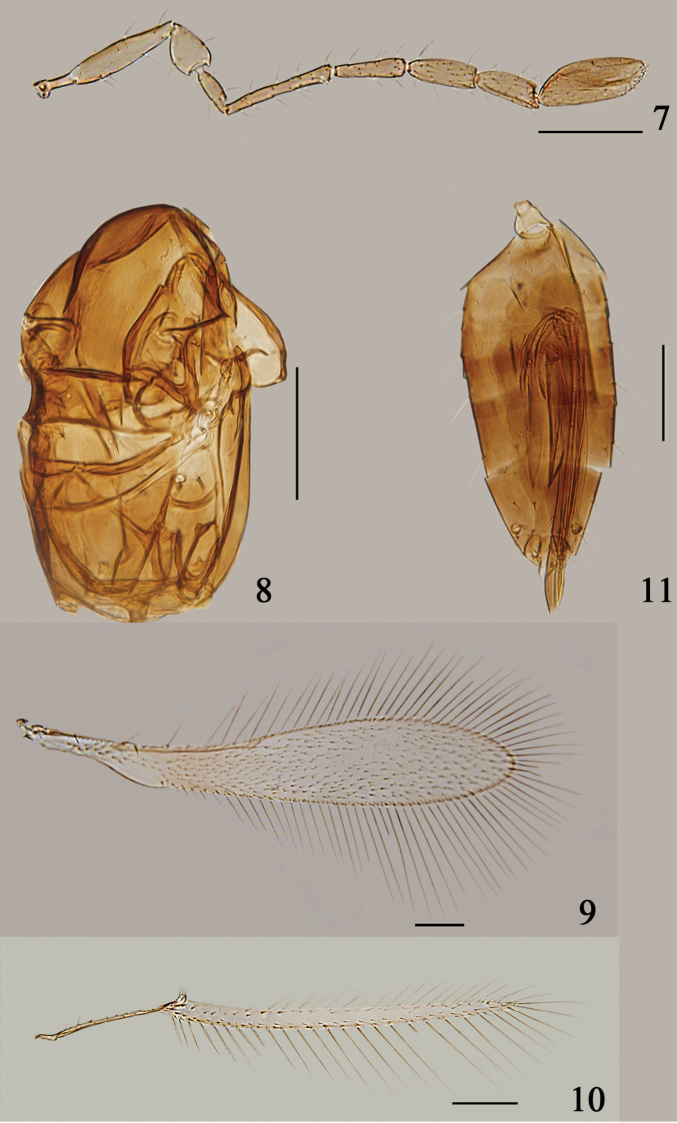
*Arescon stenopterus* sp. n., holotype female: **7** antenna **8** mesosoma, lateral **9** fore wing **10** hind wing **11** gaster, lateral. Scale bars: 100 μm.

Antenna (Fig. 7) of female with fl_2_ distinctly longer than each of fl_3_–fl_5_; clava 2.2–2.6× as long as wide, slightly shorter than scape; metanotum (Fig. 8) with dorsellum triangular; propodeum longer than scutellum; phragma broad with posterior margin nearly straight; fore wing (Fig. 9) 5.05–5.35× as long as wide, with venation extending just about half wing length; fore wing base behind submarginal vein with 2 or 3 rows of setae, with a small oval bare area behind the basal part of submarginal vein along posterior margin, and disc at the broadest part of wing with about 12 or 13 irregular rows of setae; ovipositor (Fig. 11) 1.03–1.19× as long as metatibia, distinctly exserted.

#### Description.

Female (holotype data in square brackets). Body length 730–980 [780]. Head brown with eye, ocelli, transverse trabecula and part of supraorbital trabecula dark brown. Antenna brown with radicle, scape and pedicel paler. Mesosoma brown with frenum pale yellowish brown. Wings infuscate with base of fore wing brown and largely infuscate behind marginal vein. Legs brown with trochanters and apical parts of femora paler. Metasoma brown with petiole pale yellowish brown and base of gaster and tip of ovipositor pale brown.

Head. Vertex weakly sculptured, ocelli on an almost rectangular stemmaticum; face with faint sculpture.

Antenna. Antenna (Fig. 7) sparsely setose. Radicle 0.31–0.35 [0.35]× as long as scape; scape with faint longitudinal striations, 4.2–4.9 [4.3]× as long as wide; pedicel with faint longitudinal striations, slightly shorter than fl_1_; all funicular segments much longer than wide, fl_1_–fl_3_ without mps; fl_1_ distinctly shortest, fl_2_ distinctly longest, more than twice length of fl_1_; fl_3_–fl_5_ slightly shorter and wider distad; fl_4_ with 1 mps; fl_5_ with 2 mps; clava 2.2–2.6 [2.6]× as long as wide, slightly shorter than scape, shorter than fl_4_ and fl_5_ combined, with 6 mps. Measurements (length/width): radicle 36–48 [38], scape 108–144/20–31 [113/26], pedicel 48–60/34–60 [50/38], fl_1_ 46–58/14–19 [46/17], fl_2_ 91–144/14–17 [110/14], fl_3_ 60–77/17–22 [70/19], fl_4_ 60–82/20–24 [67/26], fl_5_ 58–72/22–26 [65/26], clava 103–118/43–53 [110/43].

Mesosoma. Mesosoma (Fig. 8) with faint reticulate sculpture. Scutellum distinctly shorter than mesoscutum (57: 84); anterior scutellum subrectangular, with campaniform sensilla a little nearer to lateral margins than to each other. Metanotum with dorsellum distinctly triangular. Propodeum smooth, longer than scutellum medially. Phragma broad, with posterior margin nearly straight.

Wings. Fore wing (Fig. 9) length 950–1232 [1000], width 168–244 [188], length/width 5.05–5.35 [5.30], with venation extending about 0.46× length of wing; longest marginal setae 242–300 [242], 1.05–1.34 [1.29]× as long as greatest wing width. Fore wing base behind submarginal vein with 2 or 3 rows of setae, with a small oval bare area behind basal part of submarginal vein along posterior margin; disc at broadest part of wing with 12 or 13 irregular rows of setae. Hind wing (Fig. 10) length 718–990 [750], width 26–43 [33], length/width 23–26 [23], longest marginal setae 182–212 [200], about 6–7 [6]× as long as greatest wing width.

Metasoma. Metasoma (Fig. 11) distinctly longer than mesosoma. Petiole short, trapezoidal. Ovipositor length 300–410[320], distinctly exserted, 1.03–1.19 [1.16]× as long as metatibia (260–400 [275]).

**Figures 12–16.**
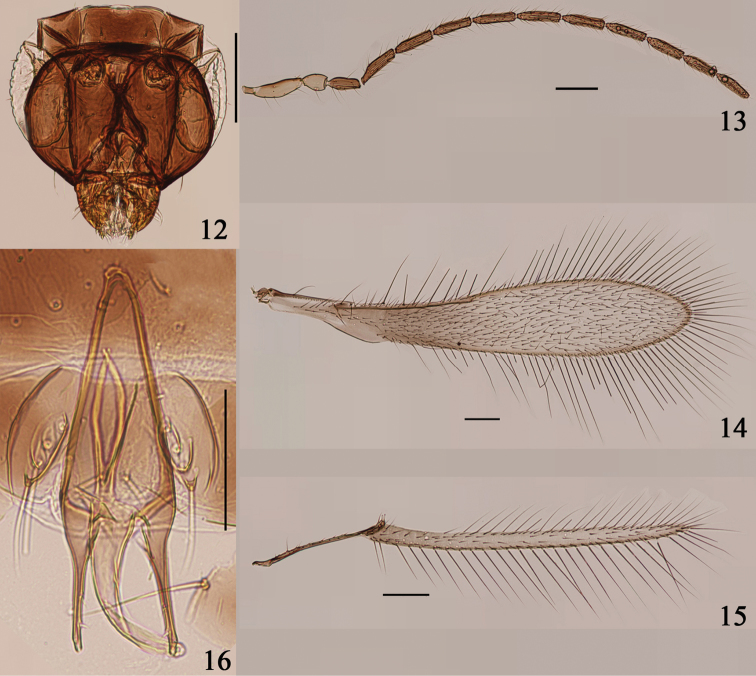
*Arescon stenopterus* sp. n., paratype male: **12** head, dorsal **13** antenna **14** fore wing **15** hind wing **16** genitalia. Scale bars: 100 μm.

**Male.** Head (Fig. 12) width 211. Antenna as in Fig. 13. Measurements (length): scape 115, pedicel 55, fl_1_ 74, fl_2_ 103, fl_3_ 96, fl_4_ 91, fl_5_ 98, fl_6_ 101, fl_7_ 98, fl_8_ 98. Fore wing (Fig. 14) length 1175, width 210, length/width 5.6, longest marginal setae 260, 1.24× as long as greatest wing width. Hind wing (Fig. 15) length 900, width 36, length/width 25, longest marginal setae 216, 6× as long as greatest wing width. Genitalia (Fig. 16) length 154.

#### Host.

Unknown.

#### Etymology.

From Greek, stenos meaning narrow and pteron meaning wing. The specific name refers to the relatively narrow fore wing.

#### Comments.

*Arescon stenopterus* sp. n., is similar to *Arescon dimidiatus* (Curtis) in that the fore wing has the venation extending just about half of the wing length and the dorsellum is distinctly triangular, but it can be distinguished from *Arescon dimidiatus* by the relatively longer fl_3_, much longer than fl_1_ (about as long as or slightly longer than fl_1_ in *Arescon dimidiatus*); relatively shorter clava, distinctly shorter than fl_4_ and fl_5_ combined (slightly longer than fl_4_ and fl_5_ combined in *Arescon dimidiatus*); and the dimensions of fore wing length and width, 5.05–5.35× as long as wide (6.5× as long as wide in *Arescon dimidiatus*).

### Arescon sparsiciliatus

Taxon classificationAnimaliaHymenopteraMymaridae

Jin & Li sp. n.

http://zoobank.org/40F855C9-3379-47C5-B7B4-3AA4C677BBD3

Figs 17–21 , 22–27

#### Holotype.

♀ (NEFU) Yunnan Province, Ruili City, Mengxiu County, 26–27.IV.2013, Xiang-Xiang Jin, Hui-Lin Han, Guo-Hao Zu, Chao Zhang, YPT.

#### Paratypes.

**5 females, 2 males.** Yunnan Province: Longchuan County, 26–27.IV.2013, Xiang-Xiang Jin, Hui-Lin Han, Guo-Hao Zu, Chao Zhang, YPT (3♀♀ 1♂, NEFU); Puer City, Lancang County, 19–20.IV.2013, Xiang-Xiang Jin, Hui-Lin Han, Guo-Hao Zu, Chao Zhang, YPT (1♀, NEFU); Mengla County, Menglun Town, 13.II. 2014, Hui-Lin Han, Guo-Hao Zu, Zhong-Ping Xiong, sweeping (1♀ 1♂, NEFU).

#### Diagnosis.

**Figures 17–21.**
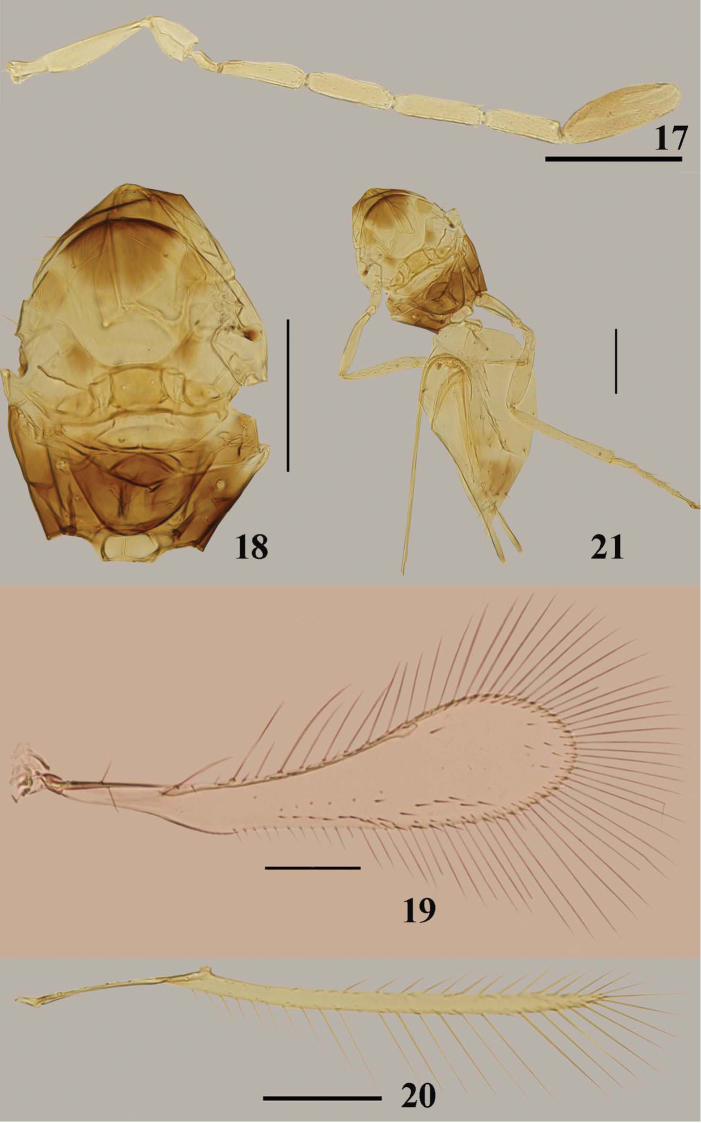
*Arescon sparsiciliatus* sp. n., holotype female: **17** antenna **18** mesosoma, dorsal **19** fore wing **20** hind wing **21** body, dorsal. Scale bars: 100 μm.

Antenna (Fig. 17) of female with fl_2_–fl_5_ almost subequal in length; clava 2.67–3.29× as long as wide, shorter than fl_4_ and fl_5_ combined; metanotum (Fig. 18) with dorsellum rhomboidal; propodeum shorter than scutellum; phragma with posterior margin narrowly rounded; fore wing (Fig. 19) 3.94–4.10× as long as wide, with venation extending about 0.7× length of wing; disc nearly asetose, only with a line along apical and posterior margins of wing, 1 or 2 irregular rows of setae near posterior margin and several scattered setae distally; ovipositor (Fig. 21) about 1.6–1.9× as long as metatibia, distinctly exserted.

#### Description.

Female (holotype data in square brackets). Body length 640–700 [655]. Head dark yellowish brown with eyes, ocelli, and transverse trabecula black; mandible brown. Antenna except clava pale brown, clava brown. Mesosoma mostly yellow with middle part of pronotum, about anterior half of mesoscutum except laterally, a small spot on tegula, middle part of metanotum, and propodeum largely except anterior lateral corner, dark brown; anterior internal part of axilla and anterior scutellum pale brown to yellowish brown. Wings uniformly infuscate with venation brown. Legs pale brown with tips of apical tarsomere of all legs brown. Metasoma pale brown with tip of gaster brown.

Head. Vertex and face with faint reticulate sculpture.

Antenna. Antenna (Fig. 17) sparsely setose. Radicle 0.24–0.31 [0.31]× as long as scape; scape with faint longitudinal striations, 3.63–4.67 [4.42]× as long as wide; pedicel with faint longitudinal striations, 1.6–1.9 [1.6]× as long as wide, longer than fl_1_; all funicular segments much longer than wide, fl_1_ distinctly shortest, without mps; fl_2_–fl_5_ each with 2 mps; fl_2_ slightly shorter than fl_3_; fl_3_ about as long as fl_4_, slightly longer than fl_5_; clava 2.67–3.29 [2.76]× as long as wide, slightly longer than scape, shorter than fl_4_ and fl_5_ combined, with 7 mps. Measurements (length/width): radicle 24–29 [26], scape 84–106/19–28 [84/19], pedicel 38–46/24–26 [38/24], fl_1_ 19–24/10 [24/10], fl_2_ 50–77/17 [62/17], fl_3_ 62–72/17 [65/17], fl_4_ 65–74/17 [65/17], fl_5_ 57–67/18 [60/18], clava 91–110/29–36 [91/33].

Mesosoma (Fig. 18). Mesoscutum longitudinally striate. Scutellum with faint reticulate sculpture distinctly shorter than mesoscutum (27: 45), with campaniform sensilla much nearer to lateral margins than to each other. Metanotum with dorsellum rhomboidal. Propodeum smooth, shorter than scutellum. Phragma with posterior margin narrowly rounded.

Wings. Fore wing (Fig. 19) length 535–560 [535], width 130–142 [135], length/width 3.94–4.10 [3.96], with venation extending 0.7× length of wing; longest marginal setae 144–175 [175], 1.06–1.30 [1.30]× as long as greatest wing width. Discal setation very sparse, only with a line along distal and posterior margins of wing, 1 or 2 irregular rows along near posterior margin, about 5–8 setae scattered on the distal part of wing and sometimes 1–5 seta(e) near stigmal vein. Hind wing (Fig. 20) length 475–530 [475], width 19, length/width 25–28 [25], longest marginal setae 101–119 [119], 5.3–6.3 [6.3]× as long as greatest wing width.

Metasoma. Metasoma (Fig. 21) distinctly longer than mesosoma. Petiole transverse. Ovipositor (340–400 [355]) about 1.6–1.9 [1.8]× as long as metatibia (194–204 [203]), distinctly exserted.

**Male.** Body length 640. Antenna (Fig. 23). Measurements (length): scape 74, pedicel 36, fl_1_ 48, fl_2_ 62, fl_3_ 60, fl_4_ 58, fl_5_ 58, fl_6_ 55, fl_7_ 55, fl_8_ 53, fl_9_ 53, fl_8_ 50, fl_9_ 48. Fore wing (Fig. 25) length 600, width 158, length/width 3.8, longest marginal setae 166, 1.05× as long as greatest wing width. Hind wing (Fig. 26) length 550, width 19, length/width 29, longest marginal setae 110, 5.79× as long as greatest wing width.

**Figures 22–27.**
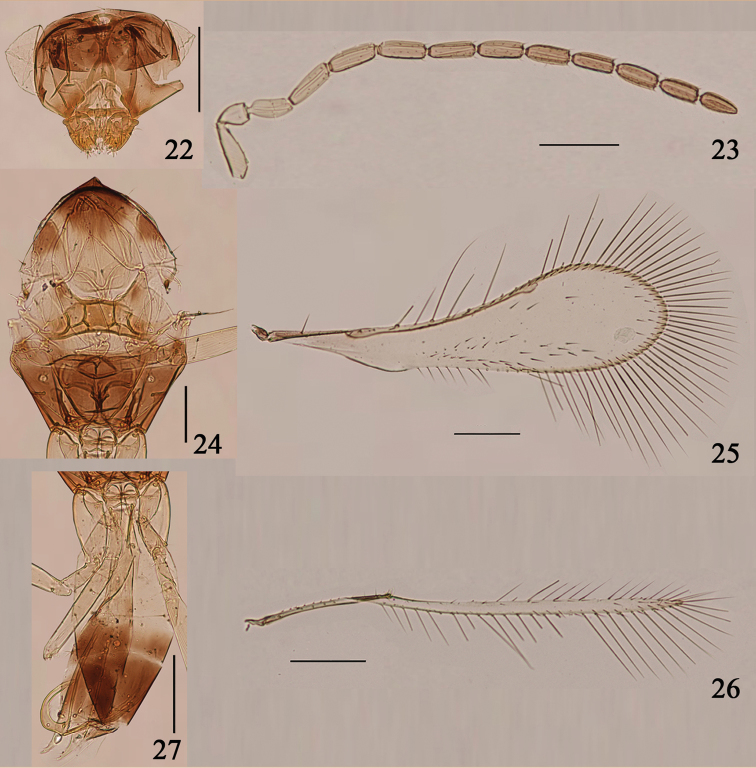
*Arescon sparsiciliatus* sp. n., paratype male: **22** head, dorsal **23** antenna **24** mesosoma, dorsal **25** fore wing **26** hind wing **27** gaster, lateral. Scale bars: 100 μm.

#### Comments.

*Arescon sparsiciliatus* sp. n. is similar to *Arescon zenit* in that fore wing venation extends almost 3/4 of the wing length and fl_2_–fl_5_ are almost subequal in length, but can be distinguished from *Arescon zenit* by the relatively more sparsely setose fore wing (more densely setose in *Arescon zenit*); relatively wider fore wing, at most 4.1× as long as wide (about 6.7× as long as wide in *Arescon zenit*); the longest marginal setae relatively shorter, at most 1.3× greatest wing width (over 2× greatest wing width in *Arescon zenit*).

## Supplementary Material

XML Treatment for Arescon gaoligongensis

XML Treatment for Arescon stenopterus

XML Treatment for Arescon sparsiciliatus
